# Hippocampal Transcriptome-Wide Association Study Reveals Correlations Between Impaired Glutamatergic Synapse Pathway and Age-Related Hearing Loss in BXD-Recombinant Inbred Mice

**DOI:** 10.3389/fnins.2021.745668

**Published:** 2021-11-17

**Authors:** Tingzhi Deng, Jingjing Li, Jian Liu, Fuyi Xu, Xiaoya Liu, Jia Mi, Jonas Bergquist, Helen Wang, Chunhua Yang, Lu Lu, Xicheng Song, Cuifang Yao, Geng Tian, Qing Yin Zheng

**Affiliations:** ^1^Precision Medicine Research Center, School of Pharmacy, Binzhou Medical University, Yantai, China; ^2^Department of Otorhinolaryngology-Head and Neck Surgery, Yantai Yuhuangding Hospital, Qingdao University, Yantai, China; ^3^Second Clinical Medical College, Binzhou Medical University, Yantai, China; ^4^Department of Plastic Surgery, Shandong Provincial Qianfoshan Hospital, The First Affiliated Hospital of Shandong First Medical University, Jinan, China; ^5^Department of Genetics, Genomics and Informatics, The University of Tennessee Health Science Center, Memphis, TN, United States; ^6^Department of Chemistry—BMC, Analytical Chemistry and Neurochemistry, Uppsala University, Uppsala, Sweden; ^7^Department of Medical Biochemistry and Microbiology, BMC, Uppsala University, Uppsala, Sweden; ^8^Department of Otolaryngology-Head and Neck Surgery, Case Western Reserve University, Cleveland, OH, United States

**Keywords:** hearing loss, systems genetics, cognitive dysfunction, glutamine synthetase (Gls), gene network, BXD mice strain, transcriptome-wide association study (TWAS)

## Abstract

Age-related hearing loss (ARHL) is associated with cognitive dysfunction; however, the detailed underlying mechanisms remain unclear. The aim of this study is to investigate the potential underlying mechanism with a system genetics approach. A transcriptome-wide association study was performed on aged (12–32 months old) BXD mice strains. The hippocampus gene expression was obtained from 56 BXD strains, and the hearing acuity was assessed from 54 BXD strains. Further correlation analysis identified a total of 1,435 hearing-related genes in the hippocampus (*p* < 0.05). Pathway analysis of these genes indicated that the impaired glutamatergic synapse pathway is involved in ARHL (*p* = 0.0038). Further gene co-expression analysis showed that the expression level of glutamine synthetase (*Gls*), which is significantly correlated with ARHL (*n* = 26, *r* = −0.46, *p* = 0.0193), is a crucial regulator in glutamatergic synapse pathway and associated with learning and memory behavior. In this study, we present the first systematic evaluation of hippocampus gene expression pattern associated with ARHL, learning, and memory behavior. Our results provide novel potential molecular mechanisms involved in ARHL and cognitive dysfunction association.

## Introduction

Hearing loss and cognitive impairment are two associated major concerns in aging populations. It is estimated that one-third of the aged population with hearing loss with different levels of cognitive decline and individuals with moderate-to-severe hearing loss are up to five times as likely to develop dementia (Lin et al., 2013; Stickel et al., 2021). Improving hearing with various hearing aids significantly improves cognitive functions, indicating that hearing loss may be causally related to cognitive decline (Sarant et al., 2020). Thus, understanding the molecular mechanisms between age-related hearing loss (ARHL) and cognitive impairment are essential, and may, in the long run, help prevent the development of age-related cognitive dysfunction (Uchida et al., 2019).

To illustrate the potential mechanism, studies have been conducted to reveal the association between hearing loss and central nervous system function, such as the frontal cortex and hippocampus (Yu et al., 2021). The hippocampus is the major brain region that regulates learning and memory; it is also involved in auditory working memory such as encoding and signal maintenance. Neurogenesis in the hippocampus is impaired with conductive or noise-induced hearing loss (Liu et al., 2018; Kurioka et al., 2021). Moreover, the hippocampus is known to be activated in response to recurring musical phrases while listening to music (Burunat et al., 2014). These studies suggest that auditory signals may regulate hippocampus signaling and molecular functions such as synaptic plasticity, which is chronically impaired by progressive hearing loss (Beckmann et al., 2020; Kurioka et al., 2021). These alterations can potentially be explained by the gene expression change associated with hearing loss (Christensen et al., 2009). However, to our knowledge, a systematic study of ARHL-associated hippocampal gene profiling has not yet been performed. Therefore, a gene profiling association study is in need to investigate molecular mechanisms that link ARHL and cognitive dysfunction (Hasson et al., 2013; Rasmussen et al., 2018).

Transcriptome-wide association study (TWAS) is a powerful tool to investigate the association between gene expression and traits (Civelek and Lusis, 2014; Wainberg et al., 2019). Novel susceptibility genes contributing to hearing impairment were identified with this approach (Xie et al., 2021). TWAS relies on the large-scale transcriptomic analysis from a genetic reference population (GRP). Among the current animal GRPs, the BXD mouse panel developed by the University of Tennessee Health Science Center (UTHSC) comprises more than 150 recombinant inbred (RI) strains with complementary traits. Owing to its genetic stability, the mice from the same strain can be considered as identical twins (Ashbrook et al., 2021). Thus, the data from multiple studies can be combined and used for systems genetics analysis including TWAS. In BXD strains, both ARHL and levels of cognitive decline showed significant variations (Zheng et al., 2020), which provide a unique platform to investigate the association between hearing loss and cognitive function.

The aim of this study is to identify potential mechanisms and key regulators that are involved in the association between ARHL and hippocampal gene profiles with a TWAS approach. We profiled the hippocampal gene expression and hearing acuity in aged BXD population. By comprehensive bioinformatics analysis, we identified that glutamate signaling in the hippocampal synapses is impaired with ARHL, and glutamine synthetase (Gls) is one of the key regulators involved in both hearing loss and cognitive decline with aging.

## Materials and Methods

### Animals

A total of 56 BXD strains (one male and one female for most strains) and 54 BXD strains (two male and two female for most strains) plus their parental strains are used for collecting hippocampus gene expression data and hearing screening, respectively. The age of all mice except one BXD101 mouse was between 12 and 32 months. The mice were housed in groups in a temperature- and humidity-controlled vivarium with a constant 12-h light–dark cycle with *ad libitum* access to food and water. For the hippocampal profiling, the mice were anesthetized by cervical dislocation. For the hearing screening, the mice were anesthetized with an intraperitoneal injection (IP) of ketamine, xylazine, and acepromazine at doses of 40, 5, and 1 mg/kg, respectively. The present study was carried out in accordance with the Guidelines for the Care and Use of Laboratory Animals published by the National Institutes of Health and was approved by the Animal Care and Use Committee at the University of Tennessee Health Science Center (UTHSC, Memphis, TN, United States).

### Hearing Acuity Assessment

Hearing acuity was assessed using an auditory-evoked brainstem response (ABR) (Intelligent Hearing Systems, Miami, FL, United States) as detailed previously (Zhou et al., 2006). Briefly, the mice were anesthetized with an intraperitoneal injection (IP) of ketamine, xylazine, and acepromazine at doses of 40, 5, and 1 mg/kg, respectively. The body temperature was maintained at 37–38°C. The ABRs were recorded using platinum subdermal needle electrodes inserted at the vertex (active electrode), ventrolateral to the right (reference electrode) and left (ground electrode) ears. The acoustic stimuli were tone-bursts (3-ms duration with a 1.5-ms cosine-gated rise/fall time) that were delivered through a high-frequency transducer. The stimuli were presented in a 5-dB step decrement from 80 dB sound pressure level (SPL) until the lowest intensity that could still evoked a reproducible ABR pattern was detected. If 80 dB could not evoke a reproducible ABR pattern, the stimuli were increased in a 5-dB step until the maximal SPL was reached. All the animals were tested with three frequencies (8, 16, and 32 kHz). For each frequency, the strain ABR is the mean value of individual ABRs, and the ABR thresholds from three frequencies were averaged and used as hearing acuity feature.

### Microarray Profiling

Snap frozen hippocampi from BXD mice across 56 strains were used for RNA quantification. RNA was extracted using the RNeasy mini kit (Qiagen, CA, United States) according to the instructions of the manufacturer. The 2100 Bioanalyzer (Agilent Technologies, Santa Clara, CA, United States) was used to evaluate RNA integrity and quality. Samples with RNA Integrity Numbers (RIN values) > 8.0 were analyzed on Affy MoGene1.0 ST at the UTHSC. Raw microarray data were normalized using the Robust Multichip Array (RMA) method (Chesler et al., 2005; Geisert et al., 2009; King et al., 2015; Lu et al., 2016). The expression data were then re-normalized using a modified Z score described in a previous publication (Chesler et al., 2005). Briefly, RMAs were first transformed into log2-values. Then, the data of each single array was converted to Z-scores, multiplied by 2, and a value of 8 was added. The normalized data is available on GeneNetwork^1^ under the “BXD” group and “Hippocampus mRNA” type with the identifier “UTHSC BXD Aged Hippocampus Affy MoGene1.0 ST (May 15) RMA Gene Level.”

### Behavioral Phenotypes Access

The published learning-related traits of the BXD mice used in this study were retrieved from our GeneNetwork. The detailed descriptions can be found in the previous publications (Graybeal et al., 2014; Delprato et al., 2015; Knoll et al., 2016; Neuner et al., 2019)]. The summary statistics and individual values are available on GeneNetwork under the “BXD” group, “trait and cofactors” type, and “BXD published phenotypes” dataset with their corresponding GN accession number listed in Supplementary Table 1.

### Gene Function Enrichment Analysis

WEB-based Gene SeT AnaLysis Toolkit (WebGestalt) was used to perform gene set enrichment analysis with the mouse genome reference gene set as the background (Liao et al., 2019)^2^. The over-representation of Gene Ontology (GO) was determined by the hypergeometric test. KEGG Orthology-Based Annotation System (KOBAS) was used to analyze the pathways involved in the ARHL correlated genes (Bu et al., 2021).

### Gene Co-expression Network Analysis

Gene co-expression network analysis has been widely used to explore the key genes in the gene sets. Therefore, we deployed this approach to identify the key genes from the gene set that correlated with hearing loss (Zhang and Horvath, 2005). Briefly, a network was constructed with Pearson’s correlation coefficient matrix (Supplementary Figure 1). In the network, each node stands for a gene, and the correlation coefficient was set as the edge. Binomial correlation higher than 0.3 or lower than −0.3 was defined as connected. The connection weight was calculated for each node, which is the sum of the binominal correlation coefficient connected to each node. The gene with the most connectivity and connection weight in the network can be considered as the central hub gene.

### Statistics Analysis

The gene–phenotype and gene–gene correlations were performed on the GeneNetwork (see text footnote 1) online platform by using Pearson’s correlation. A *p*-value lower than 0.05 was considered as statistically significant. A correlation coefficient higher than 0.3 or lower than −0.3 was considered as moderate correlation. To further validate the false discovery rate (FDR) of the genes-of-interest, we performed a further permutation test based on the Westfall and Young’s multiple testing procedure (Chaubey, 1993), Briefly, we randomly permuted the ABR measurements 1,000 times. For each permutation, we computed the *p*-value of Pearson’s correlation between the randomized ABR value and those transcripts that are associated with ABR. The adjusted *p*-value was determined by ranking the correlation coefficient.

## Results

### The Hearing Acuity in Aged BXD Mice Is Associated With Hippocampal Glutamatergic Synapse Pathway

**FIGURE 1:**
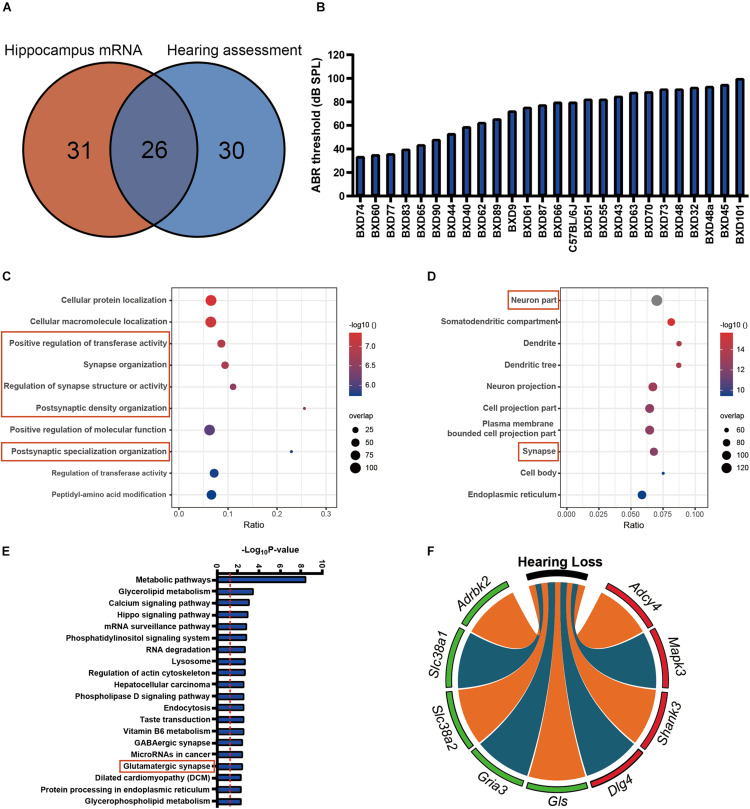
**(A)** The 26 strains with both 56 strains for auditory-evoked brainstem response (ABR) thresholds and 57 strains for hippocampus mRNA transcriptomic analysis are involved in the association analysis. **(B)** The average ABR thresholds of 26 BXD mouse strains at 2 years old age. The *x*-axis shows the BXD strains and the two parental strains. The *y*-axis shows the average ABR thresholds for 8K, 16K, and 32 kHz tone bursts were averaged as hearing acuity. **(C,D)** Bubble charts of the Gene Ontology analysis enriched for age-related hearing loss (ARHL) correlated genes. Gene over-representation analysis for Gene Ontology analysis of the ARHL correlated genes (*p*-value < 0.05 and *r* > 0.3) were performed with WebGestalt (http://www.webgestalt.org/). The *x*-axis represents an enriched ratio, and the *y*-axis represents enriched pathways/terms. The size of the dots represents the number of genes, and the color indicates the *p*-values. An enriched ratio is defined as the number of observed divided by the number of expected genes from the annotation category in the gene lists. **(E)** Glutamatergic synapse is involved in ARHL correlated pathway based on the mice hippocampus transcriptome data. The ARHL correlated genes were analyzed by KEGG. The top 20 pathways associated with the network genes were tested alongside the *p*-values calculated using a right-tailed Fisher’s exact test. **(F)** Chord-Chart of the Glutamatergic synapse pathway enriched for ARHL correlated gene. The outer color green represents negative correlation, red represents positive correlation.

A total of 26 strains that overlapped between hearing and hippocampus transcriptomic data were used for association analysis. Those strains are mainly in the similar age (from 19 to 25 months), except for BXD101 (11 months), BXD55 (27 months), and BXD45 (30 months) (Supplementary Table 2, Supplementary Figure 2, and Figure 1A). The tested animals represent a diverse degree of hearing loss determined with ABR thresholds between 33 (BXD74) and 100 dB (BXD101). The mean ABR thresholds was 71 dB SPL (Figure 1B), and the median ABR thresholds was 76 dB SPL. These data were used for the association analysis with hippocampus transcriptomic profile. In total, 1,435 genes presented significant correlation with hearing acuity (*p* < 0.05, Pearson’s correlation). With the FDR threshold of 0.05 determined by the Westfall and Young’s multiple testing procedure, equivalent to the *r*-value of 0.388, all the 1,435 transcripts were achieved significance. GO analysis of this gene set showed that four of the top 10 categories were associated with synapse organization (Figure 1C), and these genes were abundantly enriched in cellular compartment of synapse (ratio = 0.07, *p* = 6.17E−13) (Figure 1D). Further pathway analysis indicated that the glutamatergic synapse pathway is significantly enriched (ratio = 0.08, *p* = 0.0038) (Figure 1E). Nine genes that correlated with ABR thresholds were involved in this pathway, including four genes that showed positive correlation (*Adcy4*, *Mapk3*, *Shank3*, and *Dlg4*; *n* = 26, *r* > 0.4, *p* < 0.05, Pearson’s correlation), and five genes that showed negative correlation with ABR thresholds (*Adrbk2*, *Slc38a1*, *Slc38a2*, *Gria3*, and *Gls*, *n* = 26, *r* < −0.4, *p* < 0.05, Pearson’s correlation) (Figure 1F). To better clarify the gene expression correlation to each frequency, we supplemented the gene expression of synapse pathway to each of three frequencies (Supplementary Figure 3), which showed that all of those genes were significantly associated with the ABR thresholds at all three frequencies.

The mouse strains in our study are a senile population. Even though most of the strains are in similar age, there is still age variance among the strains. To exclude potential influence of such variance on the gene expression, we performed linear regression analysis between age and various target transcripts expression. The results indicated that the transcript expression variance is dominated by strain background but not age variance. Similarly, the age variation in our senile population has little effect on ABR threshold (Supplementary Tables 3, 4).

### Age-Related Hearing Loss Is Associated With Hippocampal Glutamate Receptors Expression Profiling

**FIGURE 2:**
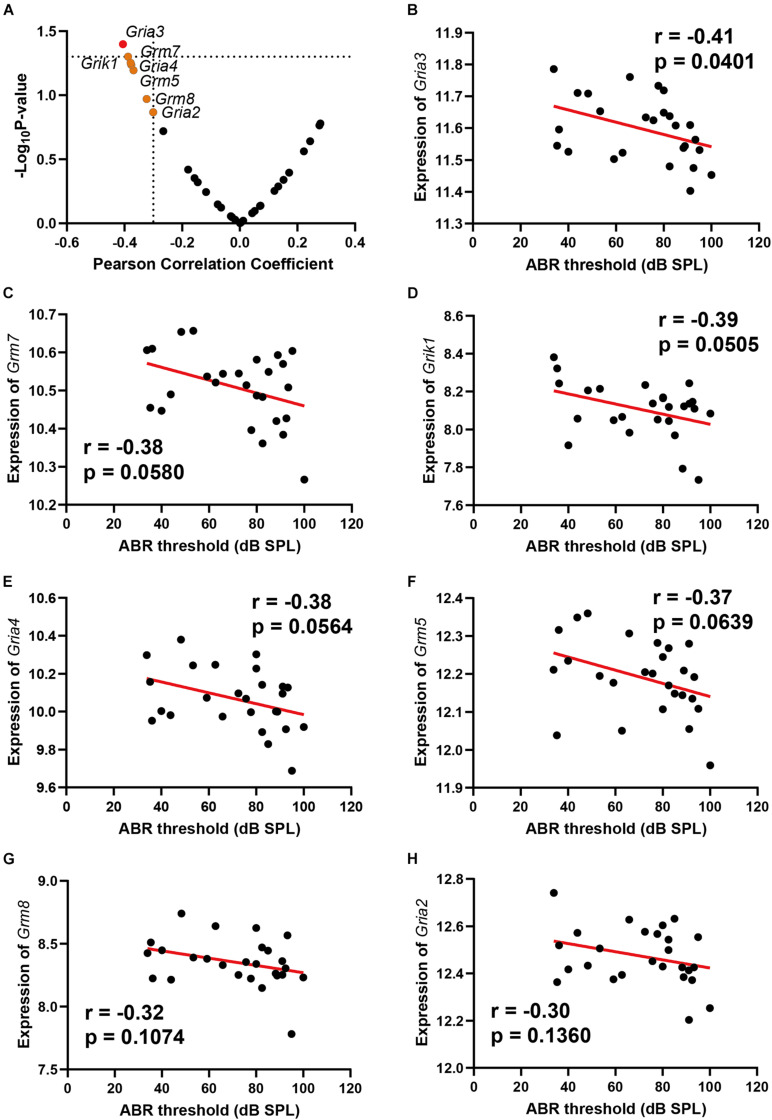
Scatterplots of the correlations of ABR threshold [dB sound pressure level (SPL)] with all glutamate receptors expression **(A)**, *Gria3* expression **(B)**, *Grm7* expression **(C)**, *Grik1* expression **(D)**, *Gria4* expression **(E)**, *Grm5* expression **(F)**, *Grm8* expression **(G)**, and *Gria2* expression **(H)**. The Pearson correlation coefficient was used to determine the relationship. Pearson correlation and *p*-value are indicated. Gene expression levels are log2 transformed and modified with Z score.

Glutamate receptors are the primary mediators of excitatory transmission in the central nervous system and play a pivotal role in learning and memory. To further investigate the glutamate receptor profiling associated with ARHL, we performed a correlation screening of ABR thresholds to 32 glutamate receptors (Figure 2A and Supplementary Table 5). Besides *Gria3*, which had the most significant correlation (*n* = 26, *r* = −0.41, *p* = 0.0401) (Figure 2B), six other receptors also showed moderate correlation to ABR thresholds (Figures 2C–H, *r* < −0.3), including *Grm7*, *Grik1*, *Gria4*, *Grm5*, *Grm8*, and *Gria2*. Notably, all these were negative correlations to ABR thresholds. These results indicated that ARHL is associated with altered glutamate receptor expression profiling in the hippocampal synapse.

### Age-Related Hearing Loss Is Associated With Reduced Glutamate Synthesis in Presynaptic Neurons

**FIGURE 3:**
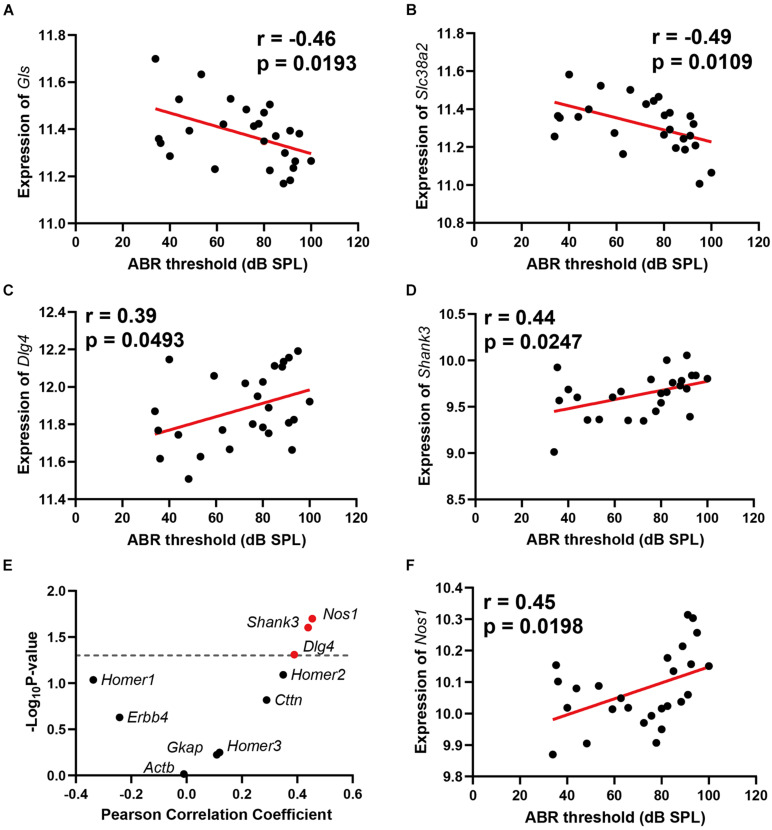
Scatterplots of the correlations of ABR threshold (dB SPL) with glutamine synthetase (*Gls)* expression **(A)**, *Slc38a2* expression **(B)**, *Dlg4* expression **(C)**, *Shank3* expression **(D)**, 10 known glutamate receptor chaperone proteins expression **(E)**, and *Nos1* expression **(F)**. The Pearson correlation coefficient was used to determine the relationship. Pearson correlation and *p*-value are indicated. Gene expression levels are log2 transformed and modified with Z score.

Two genes involved in glutamate synthesis are significantly correlated with ABR thresholds, which are *Gls* (*n* = 26, *r* = −0.46, *p* = 0.0193) and *Slc38a2* (*n* = 26, *r* = −0.49, *p* = 0.0109) (Figures 3A,B). Both genes presented negative correlation to ABR thresholds. In the glutamatergic synapse pathway, the glutamine is primarily released from astroglia cells and further imported into presynaptic neurons with glutamine receptor Slc38a2. The imported glutamine is further converted to glutamate with Gls in the presynaptic neurons. The negative correlation of these two genes with ABR thresholds indicated that glutamate synthesis is impaired in ARHL.

### Age-Related Hearing Loss Is Associated With Postsynaptic Glutamate Receptor Reorganization Regulation

Two glutamate receptor-interacting protein chaperones—Dlg4 and Shank3—were positively correlated with hearing loss (*n* = 26, *r* = 0.39, *p* = 0.0493); (*n* = 26, *r* = 0.44, *p* = 0.0247) (Figures 3C,D). Both Dlg4 and Shank3 act as chaperones to assist glutamate-receptor reorganization. Thus, we performed a further correlation screening between hearing loss and 10 known glutamate receptor chaperone proteins (Figure 3E). Besides *Dlg4* and *Shank3*, *Nos1* (*n* = 26, *r* = 0.45, *p* = 0.0198) (Figure 3F) also presented significant positive correlations with ABR thresholds. These data showed that ARHL is associated with enhanced glutamate receptor chaperone expression.

### Age-Related Hearing Loss Is Associated With Hippocampus Cyclic Adenosine Monophosphate Signaling Pathway Through Adenylate Cyclase 4

**FIGURE 4:**
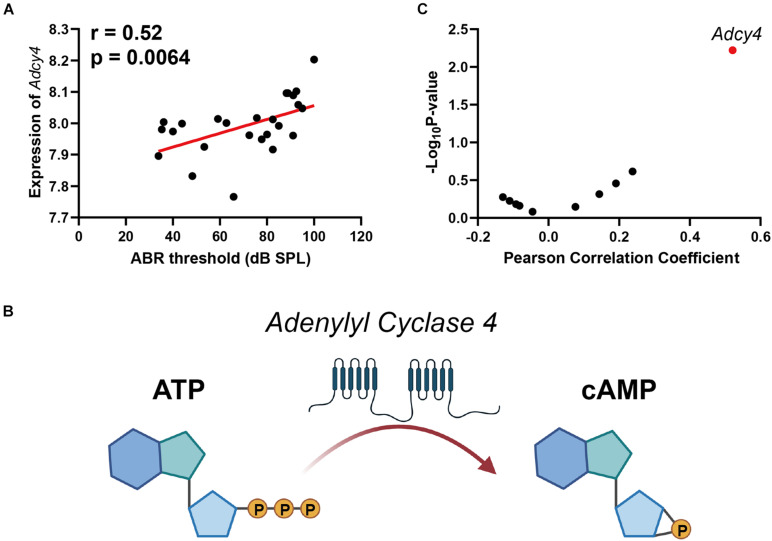
Scatterplots of the correlations of ABR threshold (dB SPL) with *Adcy4* expression **(A)**. Adenylate cyclase 4 (Adcy4) catalyzes the conversion of ATP to cyclic adenosine monophosphate (cAMP) **(B)**. Scatterplots of the correlations of ABR threshold (dB SPL) with all Adenylate Cyclase family expression **(C)**. The Pearson correlation coefficient was used to determine the relationship. Pearson correlation and *p*-value are indicated. Gene expression levels are log2 transformed and modified with Z score.

The cyclic adenosine monophosphate (cAMP) signaling is a critical second messenger signaling in the glutamatergic synapse pathway. Our correlation analysis indicated that the gene expression of *Adcy4* is significantly correlated with ABR thresholds (*n* = 26, *r* = 0.52, *p* = 0.0064) (Figure 4A). cAMP is synthesized by adenylate cyclase (Figure 4B), which is a protein family including 10 different members. Thus, a correlation screen was performed between ABR thresholds and AC family members (Figure 4C). Among the AC family, only *Adcy4* presented a significant correlation with ABR thresholds, suggesting that ARHL is associated with cAMP signaling pathway through Adcy4.

### Gene Co-expression Network Analysis Suggests That Glutamine Synthetase Is a Key Regulator Gene in Glutamate Pathway

**FIGURE 5:**
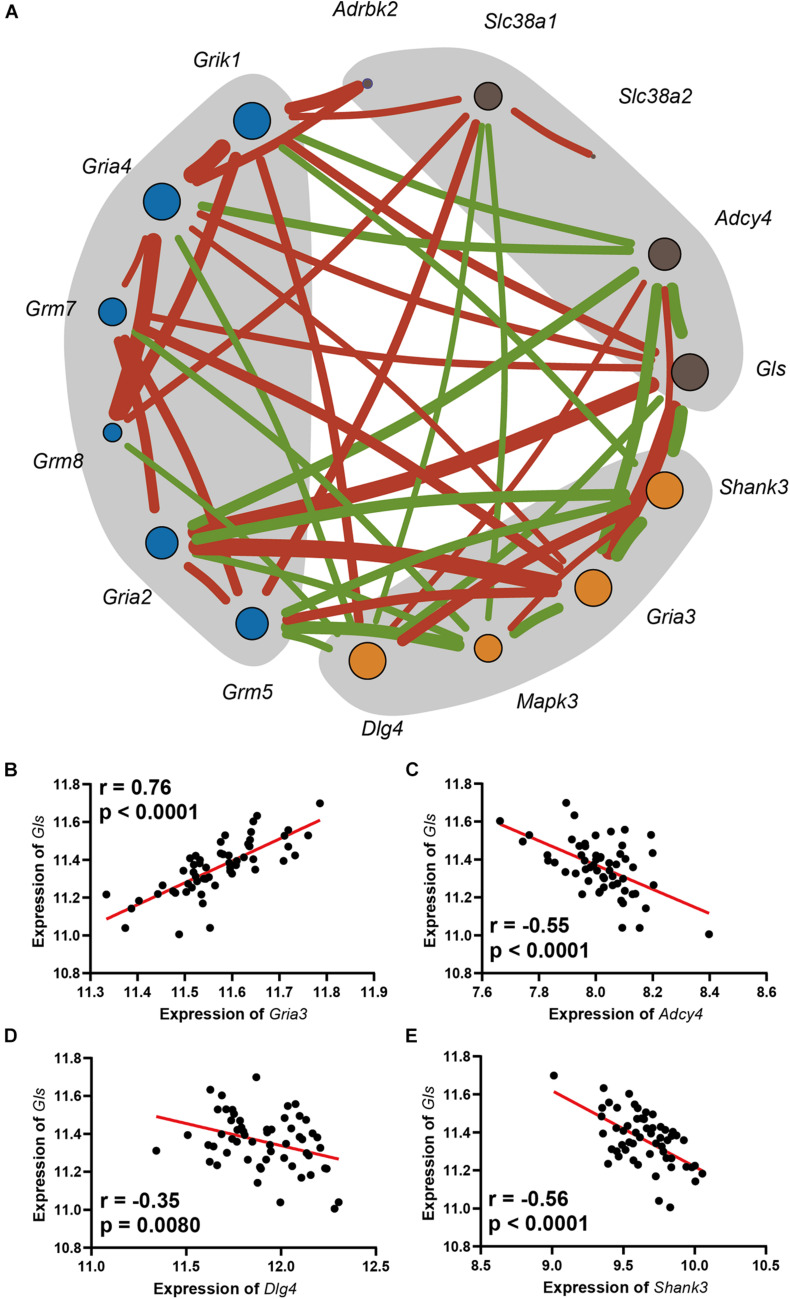
**(A)** The network includes the genes enriched in the Glutamatergic pathway, as well as the glutamate receptors that were mapped. The orange dots are in postsynapse, the blue dots are the glutamate receptors with moderate correlation with ARHL, and the dark gray dots is in presynapse, the size of the point indicates its connection degree, the red line indicates a significant positive correlation, the green line indicates a significant negative correlation, and the thickness of the line indicates the value of the correlation. Scatterplots of the correlations of *Gls* expression with *Gria3* expression **(B)**, *Adcy4* expression **(C)**, *Dlg4* expression **(D)**, *Shank3* expression **(E)**. The Pearson correlation coefficient was used to determine the relationship. Pearson correlation and *p*-value are indicated. Gene expression levels are log2 transformed and modified with Z score.

A gene co-expression network was constructed based on nine hearing loss-associated genes in the synaptic signaling pathway. Among these genes, Gls showed the highest connectivity of eight and an average connection weight of 0.49 as a central node (Figure 5A and Supplementary Tables 6, 7). Notably, *Gls* presented a significant correlation with five other hub genes; *Gria3* (*n* = 26, *r* = 0.76, *p* < 0.0001), *Adcy4* (*n* = 26, *r* = −0.55, *p* < 0.0001), *Dlg4* (*n* = 26, *r* = −0.35, *p* = 0.008), and *Shank3* (*n* = 26, *r* = −0.56, *p* < 0.0001) (Figures 5B–E). Taken together, these results suggest that Gls is a central hub gene in the glutamate signaling network.

### Phenotype-Wide Association Showed Glutamine Synthetase Is Associated With Learning Behavior in BXD Strains

**FIGURE 6:**
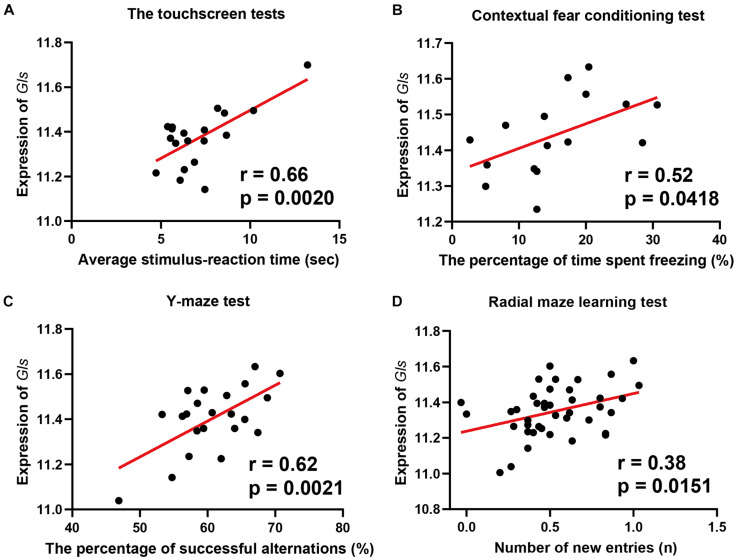
Scatterplots of the correlations of *Gls* expression with the learning latency in a touch screen test **(A)**, the percentage of time spent freezing in Contextual fear conditioning test **(B)**, the percentage of successful alternations in Y-maze test **(C)**, the number of new entries during the first 8-arm choices in an 8-arm radial maze test **(D)**. The Pearson correlation coefficient was used to determine the relationship. Pearson correlation and *p*-value are indicated. Gene expression levels are log2 transformed and modified with Z score.

To reveal the association between *Gls* expression and learning behavior, we performed a phenotype-wide association analysis between *Gls* and published phenotypes in BXD strains. The expression of *Gls* in the hippocampus is significantly correlated with 31 learning-related phenotypes (Supplementary Table 1). Briefly, the expression of *Gls* was significantly correlated to the learning latency in a touch screen test (*n* = 19, *r* = 0.66, *p* = 0.0020), the percentage of time spent freezing in contextual fear conditioning test (*n* = 16, *r* = 0.52, *p* = 0.0418), the percentage of successful alternations in the Y-maze test (*n* = 22, *r* = 0.62, *p* = 0.0021), and the number of new entries during the first 8-arm choices in an 8-arm radial maze test (*n* = 41, *r* = 0.38, *p* = 0.0151) (Figures 6A–D). These data collectively proved that the hippocampal *Gls* expression is associated with learning and memory behavior.

## Discussion

**FIGURE 7:**
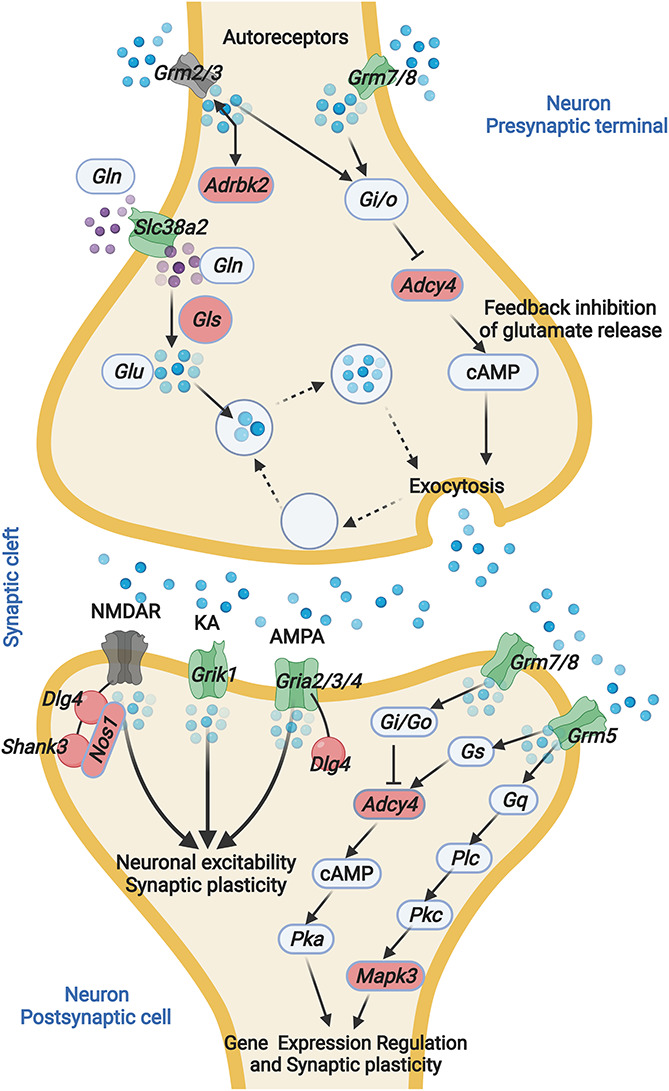
ARHL associated Glutamatergic synapse pathway mapping. Glutamate released from presynaptic terminals acts through the activation of glutamate receptors located at the postsynaptic terminal. The interaction between glutamate and glutamate receptor favors the activation of several metabolic pathways. Glutamine is converted into glutamate by glutaminase. GLN, glutamine; GLS, Glutaminase; GLU, glutamate; Grm2, glutamate metabotropic receptor 2; Grm3, glutamate metabotropic receptor 3; Grm5, glutamate metabotropic receptor 5; Grm7, glutamate metabotropic receptor 7; Grm8, glutamate metabotropic receptor 8; Slc38a2, solute carrier family 38 member 2; Adrbk2, adrenergic, beta, receptor kinase 2; Adcy4, adenylate cyclase 4; NMDAR, N-methyl-D-aspartate receptor; KA, kainate receptors; AMPA, α-amino-3-hydroxy-5-methyl-4-isoxazolepropionic acid receptor; PSD-95, postsynaptic density protein 95; Shank3, SH3 and multiple ankyrin repeat domains 3; Grik1, glutamate ionotropic receptor kainate type subunit 1; Gria2, glutamate ionotropic receptor AMPA type subunit 2; Gria3, glutamate ionotropic receptor AMPA type subunit 3; Gria4, glutamate ionotropic receptor AMPA type subunit 4. The blue sphere indicates GLU, the purple sphere indicates GLN. Green indicates negative correlation with ABR, red indicates positive correlation with ABR. Gray indicates non-significant correlation with ABR.

In this study, we present the first hippocampal gene expression profiling that associated with ARHL. It has been hypothesized that hearing loss is associated with disturbed neurogenesis and synapse plasticity (Kurioka et al., 2021). However, the detailed molecular mechanisms remain unclear. Our analysis indicates that alternation in the glutamatergic synapse signaling is associated with ARHL, including altered glutamate receptor expression and decreased glutamate synthesis (Figure 7). Further gene network analysis indicates that Gls is the key node gene in the network, and Gls expression is associated with both the hearing acuity and learning, and memory behavior. The thorough examination of hippocampus gene profiling has shed light on the potential molecular mechanism of association between hearing loss and cognitive function.

We identified that the hippocampal glutamatergic synapse pathway is associated with ARHL. This is consistent with previous reports wherein the glutamatergic synaptic connectivity in the hippocampus could be altered by noise exposure (Zhang et al., 2021). Glutamate is the excitatory neurotransmitter at many synapses in the central nervous system and has been extensively studied in relation to cognition, particularly learning and memory. The impairment of the glutamatergic synapse pathway is associated with neurodegenerative diseases such as Alzheimer’s disease and Parkinson’s disease (Findley et al., 2019). The association between glutamatergic synapse and hearing acuity provides direct support for the association between hearing loss and cognitive dysfunction.

In the hippocampal glutamatergic synapse pathway, a group of glutamate receptors showed significant correlation to hearing acuity. The expression of glutamate receptors is responsible for the excitatory drive in neuronal networks and are involved in activating downstream signaling cascades required for synaptic plasticity (Hunt and Castillo, 2012; Chater and Goda, 2014). The decreased expression of glutamate transporters was found in cognitive dysfunction diseases such as in patients with Alzheimer’s disease (Benarroch, 2010). Among the glutamate receptors, Gria3 which is a component of α-amino-3-hydroxy-5-methyl-4-isoxazolepropionic acid receptors (AMPARs), showed the most negative correlation with ABR thresholds. This is consistent with a previous report in which Gria3 mediated auditory-experience plasticity at the end bulb synapse (Clarkson et al., 2016). The activation of Gria3 can directly lead to synaptic potentiation at the CA1 hippocampal synapses (Renner et al., 2017). Our results provide extra evidence that Gria3 is a critical auditory signal mediator in the hippocampus. Moreover, other AMPARs such as Gria2 also showed moderate correlation. AMPARs have fundamental roles in both basal transmission and long-term potentiation (LTP) and long-term depression (LTD), which strongly implicates ARHL associated with learning and memory defects through altering AMPARs expression profiling.

In the gene co-expression network, Gls showed most connection with other nodes and is suggested as a key regulator. We found a significant correlation between Gls in presynapse and Gria3 in postsynapse, which implies a *trans-*synapse transmission regulation in the hippocampus. The possible mechanism is the synthesized glutamate from presynapse can regulate the Gria3 expression in postsynapse. In primary mast cells, the glutamate can induce profound upregulation of a panel of glutamate receptors including both the ionotropic type and the metabotropic type (Alim et al., 2020). This hypothesis can also be explained by the negative correlation between Gls and Adcy4. In ARHL mice, the low concentration of glutamate enhances the expression of Adcy4 and provides complementary support for the feedback inhibitory circuit in glutamate synthesis and release.

Similarly, the negative correlation between Gls and Dlg4 provides a potential explanation for the positive correlation of glutamate receptor chaperones with hearing loss. Dlg4 and Shank3 are known to increase glutamate receptor organization and enhance learning and memory function (Ehrlich et al., 2007). However, we found that glutamate receptor chaperones are positively correlated with hearing loss level. The potential reason for this is the existence of a glutamate receptor regulation feedback circuit, in which low-level glutamate release may enhance the expression of glutamate receptor scaffold proteins such as Dlg4 and Shank3, but further research is needed.

With a phenotype-wide association analysis, we showed that the Gls expression level is associated with various learning- and memory-related traits. This is consistent with previous animal model studies. The Gls knockout mice showed impaired glutamatergic synapse transmission (Masson et al., 2006), hippocampal hypometabolism in the hippocampus CA1 subregion, and were less sensitive to behavioral stimulating effects (Gaisler-Salomon et al., 2009). Taken together, these results proposed Gls as a key regulator in the glutamate synapse signaling pathway.

In this study, we performed a TWAS based on the BXD mouse panel. In this panel, hearing variation was found in different mice strains. The hearing loss level ranged from very mild hearing loss to nearly deafness. Such hearing loss occurs naturally, and hence BXD is considered as a unique animal model population to investigate ARHL. These mice strains together with the systems genetics approach can be a powerful tool in future studies on hearing loss. Nonetheless, so far, a systematic transcriptome profiling of inner ear tissues at a large population level is still lacking. Transcriptome profiling of inner ear tissues associated with aging at a population level would provide further knowledge about ARHL.

Although we focused on hippocampal glutamate synapse signaling in the current study, the gene profiling suggested more pathways such as GABA, calcium-signaling pathways, and several metabolic pathways are involved in the association. Given the complexity and heterogeneity of those two disorders, how those pathways involved in the ARHL associated cognitive dysfunction still need to be validated and could facilitate a better understanding of the association.

One potential limitation of this study is the ABR measurements and hippocampal transcriptomic profiling, which were generated from two independent groups of BXD mice. It may introduce potential bias in the analysis. However, due to the genetic makeup, the mice from the same strain can be considered as identical twins. Moreover, we involved relatively a large number of strains with the age range roughly aligns well between those two data sets (18–25 months old). Thus the results based on the joint analysis of those two data sets are still reliable and robust.

In summary, our study identified that the hippocampal glutamatergic synaptic impairment is associated with ARHL using a TWAS approach. These results provide the potential molecular mechanism of the association between cognition dysfunction and ARHL. Moreover, it also provides a novel research strategy in related studies.

## Data Availability Statement

The original contributions presented in the study are publicly available. This data can be found here: http://gn1.genenetwork.org/webqtl/main.py?FormID=sharinginfo&GN_AccessionId=711.

## Ethics Statement

The animal study was reviewed and approved by Animal Care and Use Committee at the University of Tennessee Health Science Center.

## Author Contributions

GT designed the study. QZ and TD conducted the experiment, including ABR, and performed the statistical analysis. LL, FX, and QZ organized the database. TD and JM established the network model and wrote the first draft of the manuscript. FX, JaL, JB, JnL, XL, and ChY wrote the sections of the manuscript. QZ, JB, XS, HW, CuY, and GT revised the manuscript. All authors contributed to manuscript revision, read, and approved the submitted version.

## Conflict of Interest

The authors declare that the research was conducted in the absence of any commercial or financial relationships that could be construed as a potential conflict of interest. The handling editor declared a past co-authorship with one of the authors, QZ.

## Publisher’s Note

All claims expressed in this article are solely those of the authors and do not necessarily represent those of their affiliated organizations, or those of the publisher, the editors and the reviewers. Any product that may be evaluated in this article, or claim that may be made by its manufacturer, is not guaranteed or endorsed by the publisher.

## Footnotes

1
www.genenetwork.org

2
http://www.webgestalt.org/

## Supplementary Material

Supplementary Figure 1 Pearson correlation coefficient matrix (the key genes from the gene set that correlated with hearing loss).

Supplementary Figure 2 Violin plots of the strain ages (month) at the ABR testing and hippocampus sampling.

Supplementary Figure 3 Heatmap of the Pearson correlation coefficient between glutamatergic synapse pathway genes and ABR thresholds.
