# Supplementary figures and images for: Hippocampal Transcriptome-Wide Association Study Reveals Correlations Between Impaired Glutamatergic Synapse Pathway and Age-Related Hearing Loss in BXD-Recombinant Inbred Mice
Source: Front Neurosci. 2021 Nov 17;15:745668. doi: 10.3389/fnins.2021.745668 (PMC8636065; doi:10.3389/fnins.2021.745668)

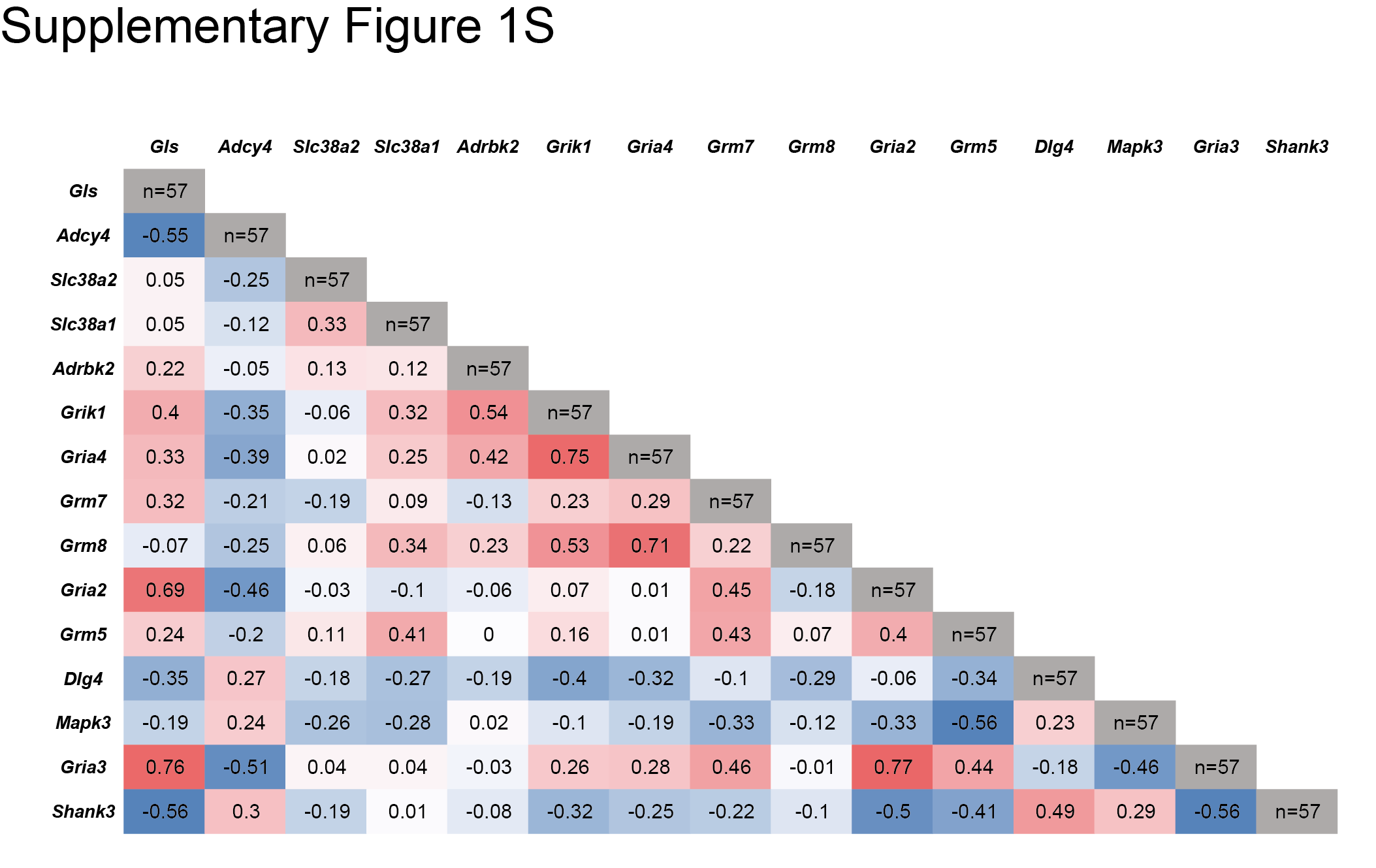

Supplement: Supplementary Figure 1 — Pearson correlation coefficient matrix (the key genes from the gene set that correlated with hearing loss). [file Image_1.TIF]

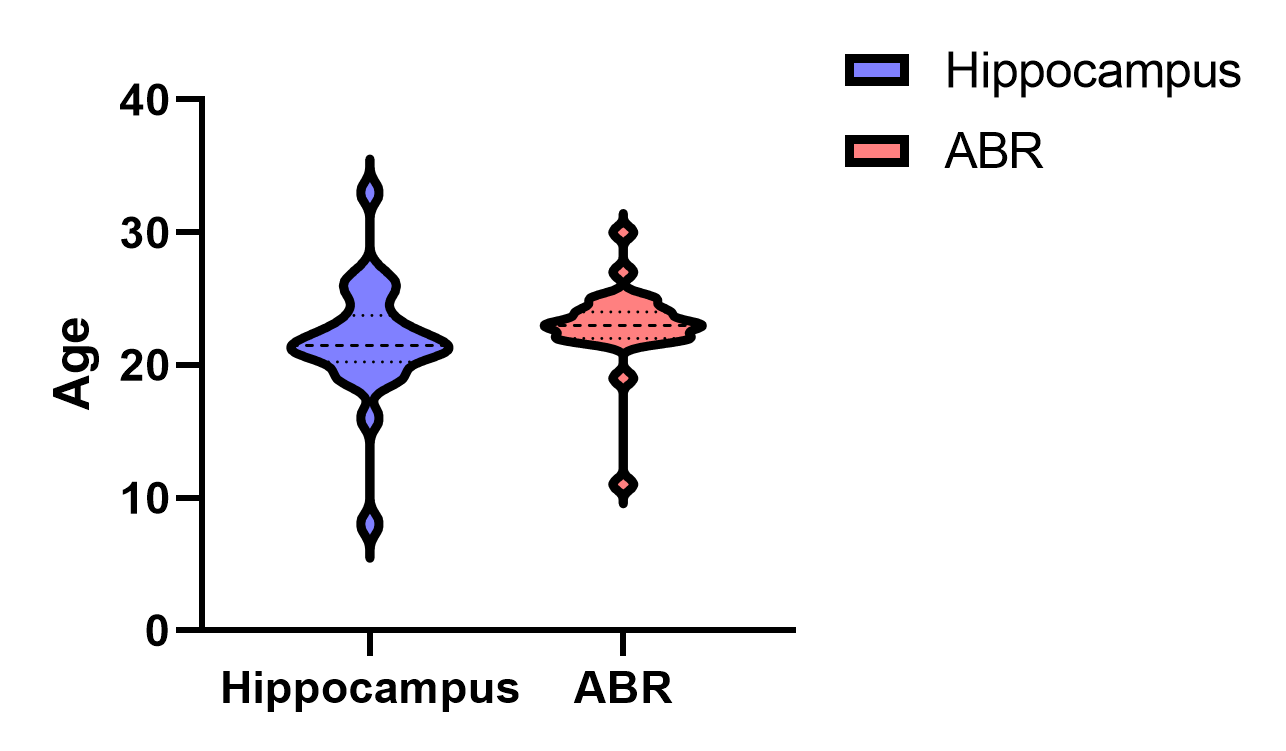

Supplement: Supplementary Figure 2 — Violin plots of the strain ages (month) at the ABR testing and hippocampus sampling. [file Image_2.TIF]

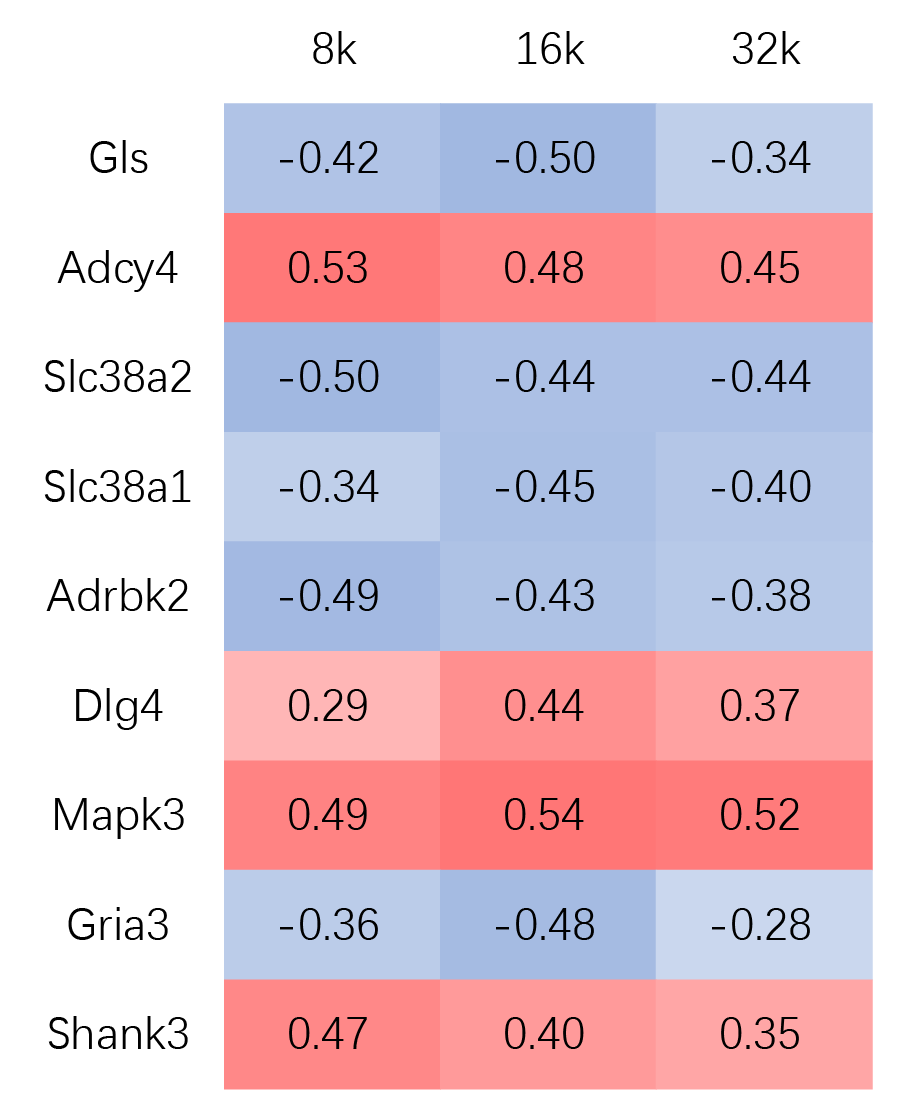

Supplement: Supplementary Figure 3 — Heatmap of the Pearson correlation coefficient between glutamatergic synapse pathway genes and ABR thresholds. [file Image_3.TIF]
