# Supplementary material for: Hippocampal Transcriptome-Wide Association Study Reveals Correlations Between Impaired Glutamatergic Synapse Pathway and Age-Related Hearing Loss in BXD-Recombinant Inbred Mice
Source: Front Neurosci. 2021 Nov 17;15:745668. doi: 10.3389/fnins.2021.745668 (PMC8636065; doi:10.3389/fnins.2021.745668)
Supplement: Supplementary file 4 [file Table_1.DOCX]

**Table 1S.** The expression of *Gls* in hippocampus is significantly correlated with 31 learning related phenotypes.

| **Record** | **Phenotype** | | **Sample r** | **Sample p(r)** |
| --- | --- | --- | --- | --- |
| **17502[1]** | | Central nervous system, behavior, learning and memory: number of new entries during the first 8-arm choices minus 5.3 in an 8-arm radial maze test on days 3 to 5 in males at 11 to 13 weeks of age [n] | 0.377023 | 0.014497 |
| **17500[1]** | | Central nervous system, behavior, learning and memory: number of new entries during the first 8-arm choices minus 5.3 in an 8-arm radial maze test on day 4 in males at 11 to 13 weeks of age [n] | 0.354301 | 0.022429 |
| **17499[1]** | | Central nervous system, behavior, learning and memory: number of new entries during the first 8-arm choices minus 5.3 in an 8-arm radial maze test on day 3 in males at 11 to 13 weeks of age [n] | 0.337667 | 0.030271 |
| **16221[2]** | | Central nervous system, behavior, learning and memory: Reversal learning reaction time to nose-poke stimuli using a touchscreen assay averaged across all reversal learning trials (Figure S4C) in 3-month-old males [sec] | 0.663062 | 0.001408 |
| **16216[2]** | | Central nervous system, behavior, learning and memory: Reversal learning using a touchscreen assay, number of errors during early reversal learning when performance was at or below 50% accuracy (see Figure 5C) in 3-month-old males [n] | 0.599573 | 0.005607 |
| **16219[2]** | | Central nervous system, behavior, learning and memory: Reversal learning correction errors using a touchscreen assay during the early phase trials when performance was at or below 50% accuracy (Figure 5D) in 3-month-old males [n] | 0.565277 | 0.010401 |
| **16211[2]** | | Central nervous system, behavior, learning and memory: Visual discrimination learning using a touchscreen assay, mean magazine latency (time to retrieve reward) across all discrimination learning sessions (see Figure S4B) in 3-month-old males [sec] | 0.518278 | 0.017953 |
| **16227[2]** | | Central nervous system, behavior, learning and memory: Extinction of a learned stimulus using a touchscreen assay, food pellet magazine latency for a non-rewarded but well learned instrumental response in 3-month-old males (supplementary trait data not in | -0.54116 | 0.018964 |
| **16218[2]** | | Central nervous system, behavior, learning and memory: Reversal learning correction errors using a touchscreen assay, means across all reversal learning trials (Figure 5B ) in 3-month-old males [n] | 0.506443 | 0.025632 |
| **16215[2]** | | Central nervous system, behavior, learning and memory: Reversal learning errors using a touchscreen assay across all reversal learning sessions (see Figure 5A) in 3-month-old males [n] | 0.471456 | 0.040583 |
| **17964** | | Central nervous system, behavior, learning and memory: Object-location paired-associates learning using a touchscreen assay in 3 to 9-month old females, average correct touch latency (time between visual stimuli being displayed on screen and mouse touchin | 0.595177 | 0.010304 |
| **17957** | | Central nervous system, behavior, learning and memory: Object-location paired-associates learning using a touchscreen assay in 3 to 9-month-old females, average number of correction trials per trial in last approximately 180 training trials, where lower v | 0.589938 | 0.011237 |
| **17956** | | Central nervous system, behavior, learning and memory: Object-location paired-associates learning using a touchscreen assay in 3 to 9-month-old females, overall percentage of trials correct in last approximately 180 training trials, where higher values me | -0.57537 | 0.014179 |
| **17944** | | Central nervous system, behavior, learning and memory: Object-location paired-associates learning using a touchscreen assay in 3 to 9-month-old females, overall percentage of trials correct in first approximately 360 training trials, where higher values m | -0.52191 | 0.030289 |
| **17965** | | Central nervous system, behavior, learning and memory: Object-location paired-associates learning using a touchscreen assay in 3 to 9-month old females, average incorrect touch latency (time between visual stimuli being displayed on screen and mouse touch | 0.515694 | 0.032806 |
| **17943** | | Central nervous system, behavior, learning and memory: Object-location paired-associates learning using a touchscreen assay in 3 to 9-month-old females, total sessions required to complete approximately 1800 training trials, where higher values means infe | 0.504576 | 0.03769 |
| **17952** | | Central nervous system, behavior, learning and memory: Object-location paired-associates learning using a touchscreen assay in 3 to 9-month-old females, overall percentage of trials correct in first approximately 1800 training trials, where higher values | -0.49862 | 0.040517 |
| **17472** | | Central nervous system, behavior, learning and memory: Object-location paired-associates learning using a touchscreen assay in 3 to 9-month-old females, percentage of trials correct in the final 180 trials at the end of training, where higher values means | -0.52163 | 0.0304 |
| **17470** | | Central nervous system, behavior, learning and memory: Object-location paired-associates learning using a touchscreen assay in 3 to 9-month-old females, overall percentage of trials correct in approximately 1800 training trials, where higher values means | -0.50608 | 0.036999 |
| **18070[3]** | | Central nervous system, behavior, learning and memory: Fear conditioning expression, average freezing response time during the first three (USC) or one (NIH) CS presentation(s) in adult males (USC) and adult males and females (NIH) [%]. | -0.40965 | 0.004797 |
| **20575[4]** | | Central nervous system, behavior, learning and memory: Performance on the y-maze as measured by % of total arm entries that were unsuccessful spontaneous alternations; known as alternating arm returns (also can be used as an error index). This data is str | -0.62326 | 0.00109 |
| **20562[4]** | | Central nervous system, behavior, learning and memory: Performance on the y-maze as measured by % of total arm entries that were successful spontaneous alternations. This data is strain averaged across 4-month-old male and female non-transgenic littermate | 0.619783 | 0.001585 |
| **20809[4]** | | Central nervous system, behavior, learning and memory: Performance on the y-maze as measured by % of total arm entries that were successful spontaneous alternations. This data is strain averaged across 4-month-old female non-transgenic littermates from th | 0.540448 | 0.008384 |
| **20494[4]** | | Central nervous system, behavior, learning and memory: Performance on the y-maze as measured by % of total arm entries that were unsuccessful spontaneous alternations; known as alternating arm returns (also can be used as an error index). This data is str | -0.50727 | 0.010411 |
| **20637[4]** | | Central nervous system, behavior, learning and memory: Age at onset of working memory deficits; individual mice defined as "impaired" when chance performance dropped below chance (50%). This data is strain averaged across females from the AD-BXD populatio | 0.524206 | 0.011168 |
| **20822[4]** | | Central nervous system, behavior, learning and memory: Performance on the y-maze as measured by % of total arm entries that were unsuccessful spontaneous alternations; known as alternating arm returns (also can be used as an error index). This data is str | -0.54012 | 0.012713 |
| **20720[4]** | | Central nervous system, behavior, learning and memory: Age at onset of working memory deficits; individual mice defined as "impaired" when chance performance dropped below chance (50%). This data is strain averaged across males from the AD-BXD population | -0.56362 | 0.016957 |
| **20893[4]** | | Central nervous system, behavior, learning and memory: Performance on the y-maze as measured by % of total arm entries that were unsuccessful spontaneous alternations; known as alternating arm returns (also can be used as an error index). This data is str | -0.60987 | 0.018746 |
| **20658[4]** | | Central nervous system, behavior, learning and memory: Performance on the y-maze as measured by % of total arm entries that were unsuccessful spontaneous alternations; known as alternating arm returns (also can be used as an error index). This data is str | -0.44547 | 0.036794 |
| **20955[4]** | | Central nervous system, behavior, learning and memory: Contextual fear conditioning, post-shock 1 freezing - the percentage of time during the 40s period following the offset of shock #1 spent freezing. This data is strain averaged across 14-month-old male non-transgenic littermates from the AD-BXD population (Ntg-BXDs) [%] | 0.513786 | 0.040614 |
| **20096** | | Central nervous System, Behavior, Motor Learning Impairment: Improvements in performance of time [sec] in continuously adapt to constant change in acceleration (4 to 40 rpm) over 2 minutes at the postnatal ages (P)90-P120 | -0.66244 | 0.024154 |

[1] A. Delprato, B. Bonheur, M.P. Algeo, P. Rosay, L. Lu, R.W. Williams, and W.E. Crusio, Systems genetic analysis of hippocampal neuroanatomy and spatial learning in mice. Genes Brain Behav 14 (2015) 591-606.

[2] C. Graybeal, M. Bachu, K. Mozhui, L.M. Saksida, T.J. Bussey, E. Sagalyn, R.W. Williams, and A. Holmes, Strains and stressors: an analysis of touchscreen learning in genetically diverse mouse strains. PLoS One 9 (2014) e87745.

[3] A.T. Knoll, L.R. Halladay, A.J. Holmes, and P. Levitt, Quantitative Trait Loci and a Novel Genetic Candidate for Fear Learning. J Neurosci 36 (2016) 6258-68.

[4] S.M. Neuner, S.E. Heuer, M.J. Huentelman, K.M.S. O'Connell, and C.C. Kaczorowski, Harnessing Genetic Complexity to Enhance Translatability of Alzheimer's Disease Mouse Models: A Path toward Precision Medicine. Neuron 101 (2019) 399-411 e5.

**Table 2S.** lists of the strain ages (month) at the ABR testing and hippocampus sampling.

| **Strains** | **ABR** | **Hippocampus** |
| --- | --- | --- |
| BXD101 | 11 | 8 |
| BXD32 | 23 | 19 |
| BXD40 | 23 | 22 |
| BXD43 | 22 | 21 |
| BXD44 | 22 | 19 |
| BXD45 | 30 | 22 |
| BXD48 | 22 | 25 |
| BXD51 | 22 | 21 |
| BXD55 | 27 | 26 |
| BXD60 | 23 | 21 |
| BXD61 | 25 | 23 |
| BXD62 | 23 | 20 |
| BXD63 | 22 | 33 |
| BXD65 | 24 | 24 |
| BXD66 | 25 | 19 |
| BXD70 | 23 | 26 |
| BXD73 | 25 | 21 |
| BXD74 | 23 | 27 |
| BXD77 | 24 | 22 |
| BXD83 | 24 | 21 |
| BXD87 | 24 | 23 |
| BXD89 | 22 | 21 |
| BXD9 | 19 | 22 |
| BXD90 | 23 | 16 |

**Table 3S.** Results of the linear regression for the ABR thresholds with age as the confounder.

| **ABR Frequency** | **Estimate Std.** | **Error** | **t value** | **Pr(>\|t\|)** |
| --- | --- | --- | --- | --- |
| 8kHz | -0.4776 | 1.5907 | -0.300 | 0.7668 |
| 16kHz | -0.9164 | 1.6373 | -0.560 | 0.5813 |
| 32kHz | -1.033 | 1.147 | -0.900 | 0.37770 |

**Table 4S.** Results of the linear regression for the gene expression with age as the confounder.

| **Gene** | **Estimate Std.** | **Error** | **t value** | **Pr(>\|t\|)** |
| --- | --- | --- | --- | --- |
| *Gls* | 0.008005 | 0.008732 | 0.917 | 0.369 |
| *Dlg4* | -0.00089 | 0.012993 | -0.069 | 0.946 |
| *Gria3* | 0.007791 | 0.006555 | 1.189 | 0.247 |
| *Shank3* | 0.001734 | 0.015706 | 0.11 | 0.913 |
| *Mapk3* | -0.00859 | 0.00723 | -1.188 | 0.247 |
| *Adcy4* | -0.0096 | 0.005608 | -1.712 | 0.101 |
| *Slc38a2* | 0.003953 | 0.009038 | 0.437 | 0.666 |
| *Slc38a1* | 0.00535 | 0.007316 | 0.731 | 0.472 |
| *Adrbk2* | 0.002148 | 0.009687 | 0.222 | 0.827 |

**Table 5S.** The correlation of ABR to 32 glutamate receptors

| **Gene name** | **Pearson Correlation Coefficient** | **P-value** |
| --- | --- | --- |
| *Grik2* | -0.17925 | 0.381 |
| *Grik4* | 0.011993 | 0.954 |
| *Grik3* | 0.043195 | 0.834 |
| *Grin2d* | 0.052157 | 0.8 |
| *Grin1* | 0.152104 | 0.458 |
| *Grinl1a* | 0.071933 | 0.727 |
| *Gria3* | -0.40506 | 0.04 |
| *Grin2c* | 0.120051 | 0.559 |
| *Grina* | 0.244206 | 0.229 |
| *Gria1* | 0.000944 | 0.996 |
| *Grm5* | -0.36854 | 0.064 |
| *Grin2b* | -0.03091 | 0.881 |
| *Grip1* | -0.14561 | 0.478 |
| *Grin3b* | 0.004377 | 0.983 |
| *Grin2a* | -0.15688 | 0.444 |
| *Grm3* | -0.06477 | 0.753 |
| *Grm6* | 0.276386 | 0.172 |
| *Grid2ip* | 0.070483 | 0.732 |
| *Gria2* | -0.30037 | 0.136 |
| *Grid1* | 0.171404 | 0.402 |
| *Grm1* | -0.02702 | 0.896 |
| *Grid2* | -0.26502 | 0.191 |
| *Grik1* | -0.38751 | 0.05 |
| *Grm8* | -0.32306 | 0.107 |
| *Grin2b* | -0.117 | 0.569 |
| *Grm4* | -0.016 | 0.938 |
| *Grip2* | -0.07646 | 0.71 |
| *Grm7* | -0.37655 | 0.058 |
| *Grin3a* | 0.222613 | 0.274 |
| *Grm2* | 0.133689 | 0.515 |
| *Gria4* | -0.37869 | 0.056 |
| *Grik5* | 0.279693 | 0.166 |

**Table 6S.** Network Nodes

| id | Gene name | type.label | degree | ave weight |
| --- | --- | --- | --- | --- |
| s01 | *Gls* | presynaptic | 8 | 49.50 |
| s02 | *Adcy4* | presynaptic | 7 | 40.43 |
| s03 | *Slc38a2* | presynaptic | 1 | 33.00 |
| s04 | *Slc38a1* | presynaptic | 6 | 32.50 |
| s05 | *Adrbk2* | presynaptic | 2 | 48.00 |
| s06 | *Grik1* | suggestive | 8 | 45.13 |
| s07 | *Gria4* | suggestive | 8 | 43.63 |
| s08 | *Grm7* | suggestive | 6 | 38.00 |
| s09 | *Grm8* | suggestive | 4 | 46.75 |
| s10 | *Gria2* | suggestive | 7 | 51.43 |
| s11 | *Grm5* | suggestive | 7 | 42.71 |
| s12 | *Dlg4* | postsynapyic | 8 | 34.00 |
| s13 | *Mapk3* | postsynapyic | 6 | 37.50 |
| s14 | *Gria3* | postsynapyic | 8 | 53.00 |
| s15 | *Shank3* | postsynapyic | 8 | 42.88 |

**Table 7S.** Network Edges

| from | to | type | weight |
| --- | --- | --- | --- |
| s01 | s02 | Negative | 55 |
| s01 | s06 | Positive | 40 |
| s01 | s07 | Positive | 33 |
| s01 | s08 | Positive | 32 |
| s01 | s10 | Positive | 69 |
| s01 | s12 | Negative | 35 |
| s01 | s14 | Positive | 76 |
| s01 | s15 | Negative | 56 |
| s02 | s06 | Negative | 35 |
| s02 | s07 | Negative | 39 |
| s02 | s10 | Negative | 46 |
| s02 | s12 | Positive | 27 |
| s02 | s14 | Negative | 51 |
| s02 | s15 | Positive | 30 |
| s03 | s04 | Positive | 33 |
| s04 | s06 | Positive | 32 |
| s04 | s09 | Positive | 34 |
| s04 | s11 | Positive | 41 |
| s04 | s12 | Negative | 27 |
| s04 | s13 | Negative | 28 |
| s05 | s06 | Positive | 54 |
| s05 | s07 | Positive | 42 |
| s06 | s07 | Positive | 75 |
| s06 | s09 | Positive | 53 |
| s06 | s12 | Negative | 40 |
| s06 | s15 | Negative | 32 |
| s07 | s08 | Positive | 29 |
| s07 | s09 | Positive | 71 |
| s07 | s12 | Negative | 32 |
| s07 | s14 | Positive | 28 |
| s08 | s10 | Positive | 45 |
| s08 | s11 | Positive | 43 |
| s08 | s13 | Negative | 33 |
| s08 | s14 | Positive | 46 |
| s09 | s12 | Negative | 29 |
| s10 | s11 | Positive | 40 |
| s10 | s13 | Negative | 33 |
| s10 | s14 | Positive | 77 |
| s10 | s15 | Negative | 50 |
| s11 | s12 | Negative | 34 |
| s11 | s13 | Negative | 56 |
| s11 | s14 | Positive | 44 |
| s11 | s15 | Negative | 41 |
| s12 | s15 | Positive | 49 |
| s13 | s14 | Negative | 46 |
| s13 | s15 | Positive | 29 |
| s14 | s15 | Negative | 56 |
